# Neural stem cell-specific deletion of *Atg7* alleviates hippocampal dysfunction and neuronal alterations induced by chronic restraint stress

**DOI:** 10.1186/s13041-025-01189-8

**Published:** 2025-03-21

**Authors:** Hyeonjeong Jeong, Seongwon Choe, Seonghee Jung, Seong-Woon Yu

**Affiliations:** Department of Brain Sciences, Daegu Gyeongbuk Institute of Science and Technology (DGIST), Daegu, 42988 Republic of Korea

## Abstract

Adult hippocampal neurogenesis is inhibited by chronic psychological stress and impaired neurogenesis underlies stress-related psychological disorders. We previously reported that chronic restraint stress (CRS) evokes autophagic death of adult hippocampal neural stem cells (NSCs) while NSC-specific deletion of *Atg7* prevents death of NSCs. Examination of cognitive ability and mood regulation next day of the termination of stress showed normal hippocampal function in mice deficient of *Atg7*. However, it was not investigated whether the preservation of NSC pool alleviates hippocampal neuronal alterations. Here, we show that CRS increased c-Fos-positive, activated neurons in the granule cell layer and decreased spine density of CA3 neurons in the hippocampus, and these hippocampal neuronal deficits were prevented by NSC-specific deletion of *Atg7*. Of note, our observation was conducted right after the termination of CRS. Therefore, our results suggest that the detrimental effects of stress on hippocampal neurons can be buffered by NSCs independent of neurogenesis and NSCs are essential to the hippocampal function both through the neurogenesis-dependent developmental process and by direct regulation of neural activation.

**Supplementary Information:**

The online version contains supplementary material available at 10.1186/s13041-025-01189-8.

Hippocampus is a limbic structure vulnerable to psychological stress, due to highly enriched expression of glucocorticoid receptor [1]. Therefore, several studies have shown that chronic stress induces neuronal atrophy and alters hippocampal functions [2]. In addition, psychological stress inhibits adult hippocampal neurogenesis and impaired neurogenesis is intimately implicated in many detrimental aspects of stress, including memory deficit, mood dysregulation, anxiogenic and depressive behaviors [3]. While adult hippocampal NSCs are inflicted by stress, adult hippocampal neurogenesis inversely buffers stress-induced hippocampal maladaptation, and enhancement of adult neurogenesis attenuates the deleterious outcomes of stress [4]. Therefore, the inhibitory effects of stress on adult hippocampal neurogenesis underlies stress-induced various psychological conditions and elucidation of this unique neural process will provide clues for better therapeutic design for the treatment of stress-induced neurological disorders.

Autophagy is an evolutionarily conserved, lysosome-dependent catabolic pathway [5]. Usually, autophagy serves as pro-survival process under stress conditions including nutrient starvation or grow factor deprivation and maintains homeostasis by recycling useless or toxic cellular molecules [5]. However, there are several studies that prolonged or excessive autophagy can lead to cell death, which is called as autophagic cell death (ACD) [6]. The first authentic case of ACD in vivo was observed in *Drosophila*, where autophagy is required for caspase-independent death of midgut cells [7]. However, the physiological roles of ACD in mammals remains understudied.

We previously reported that chronic psychological stress suppresses adult hippocampal neurogenesis by inducing ACD of NSCs using different animal models of stress, such as CRS and chronic unpredictable stress [8, 9]. NSC-specific deletion of *Atg7*, a key gene in autophagy process, prevents ACD of hippocampal NSCs, and these conditional knockout mice, called *Atg7*^NSC^ cKO mice hereafter, maintain an intact number of NSCs and are resilient to stress-induced memory deficits and mood dysregulation. Since the hippocampal function remains normal in *Atg7*^NSC^ cKO mice right after chronic stress without involvement of neurogenesis, it will be an interesting question to ask whether the preservation of NSC pool also directly protects hippocampal neurons against stress. To address this question, we examined activation state and morphologic alteration of hippocampal neurons after CRS by comparing control and *Atg7*^NSC^ cKO mice.

We established modified CRS scheme from 6 h/day for 1-week to 3 h/day for 2-week, since 2-week CRS protocol induces more various behavioral deficits than 1-week CRS right after stress in mouse model [10, 11]. We generated *Atg7*^NSC^ cKO by crossing tamoxifen (TAM)-inducible Cre-ERT2 with *Nestin* gene promotor to heterozygous *Atg7* floxed (fl/+) mice. Due to developmental abnormalities of homozygous *Atg7* mice (fl/fl) with *Nestin*-Cre, we used *Atg7* mice (fl/+) to avoid long-term inhibition of basal autophagy, as we previously explained [12]. As a control, *Nestin*-Cre/ERT2 only mice with TAM injection were used and designated as Ctrl hereafter. We first examined the number of NSCs after CRS (Fig. 1A). Both of Ctrl and *Atg7*^NSC^ cKO mice showed weight loss as a hallmark of CRS (Fig. 1B) [4]. CRS induced reduction in both total NSCs (SOX2+) and proliferating NSCs (SOX2 + KI67+) in Ctrl group. However, *Atg7*^NSC^ cKO showed intact number of both NSC populations (Fig. 1C-E). There was no significant difference in the ratio of proliferating NSCs across all the groups, implying that loss of NSCs was due to cell death, not decreased proliferation (Fig. 1F). These results indicated that deletion of *Atg7* prevents death of NSCs after 2-week CRS. We then analyzed hippocampus-dependent behaviors. Arm alternation in Y-maze test was decreased in Ctrl CRS group, indicating working memory deficits (Fig. 1G). In the open field test (OFT), Ctrl CRS group spent less time in the center zone, showing increase in anxiety-like behavior (Fig. 1I). Ctrl CRS group exhibited increased immobility time in forced swimming test and decreased sucrose preference, indicating CRS induced depressive-like behaviors (Fig. 1K, L). However, these behavioral phenotypes caused by CRS were effectively prevented in *Atg7*^NSC^ cKO mice (Fig. 1G-L). All groups showed no differences in total arm entries (Y-Maze) and total distance moved (OFT), suggesting normal basal locomotive behavior (Fig. 1H, J). Taken together, cognitive deficits and mood dysregulation were diminished by preventing CRS-induced autophagic death of NSCs. Granule cells (GCs) regulates hippocampal function by mediating signaling from entorhinal cortex to CA3 as a major part of trisynaptic system [2]. The neurons in CA3 subregion receive signal from GCs through mossy fibers and severely suffers from chronic stress [2, 13]. We observed morphological changes of neuron in CA3 subregion by Golgi-Cox staining. Spine density of neuron was dramatically diminished after CRS in Ctrl mice but *Atg7*^NSC^ cKO mice did not undergo atrophy (Fig. 1M, N). Previous study revealed that stress susceptibility occurs with activation of mature GCs in ventral DG and their inhibition leads to stress resilience [14]. To demonstrate that NSCs regulates activity of GCs in DG immediately after stress, we detected c-Fos expression, a marker of neuronal activity, in GCL right after CRS. The number of c-Fos + neuron in GCL was highly increased by CRS in Ctrl, but not in *Atg7*^NSC^ cKO mice (Fig. 1O, P). Thus, inhibition of autophagy in NSCs not only preserves the NSC pool against CRS, but also attenuates CRS-inflicted neuronal overactivation and structural alterations in the hippocampus.

**Fig. 1:**
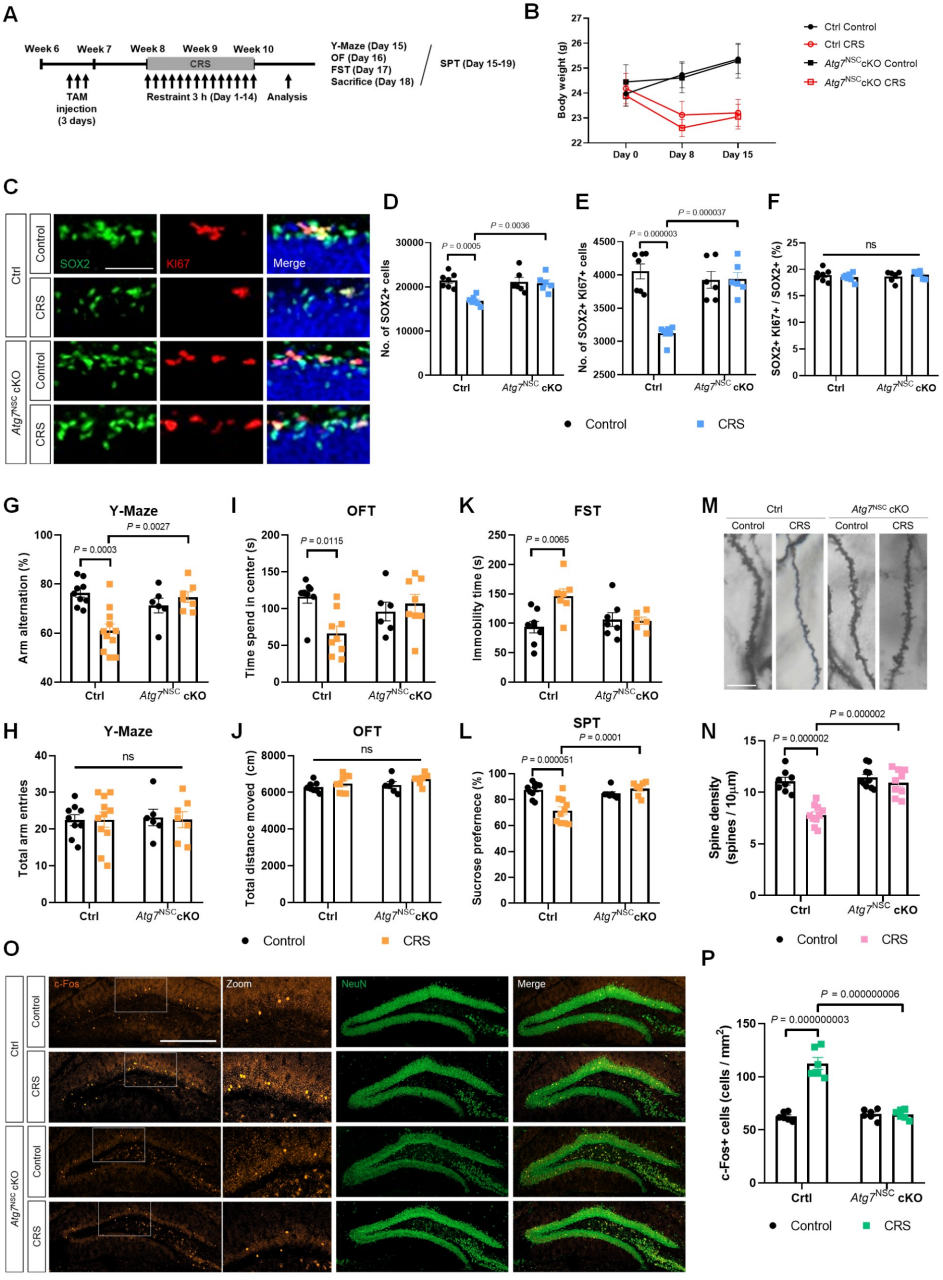
CRS-induced neuronal alterations are alleviated in *Atg7*^NSC^ cKO mice. (**A**) Timeline of the experiment. (**B**) Change of body weight (*n* = 6–7 per group). (**C**) Representative images of NSCs in dentate gyrus, Scale bar: 50 μm. (**D**-**F**) Quantification of SOX2 + and KI67 + cells (*n* = 6–7 per group). (**G**, **H**) The spontaneous arm alternation (**G**) and the number of total arm entry (**H**) in Y-Maze test. (**I**, **J**) Time spent in the center zone (**I**) and total distance moved (**J**) in in OFT. (**K**, **L**) Depression-like behavior test in FST (**K**) and SPT (**L**) (*n* = 6–10 per group). (**M**, **N**) Representative images of dendrite spines in the CA neurons by Golgi-cox staining (**M**) and quantification graph of spine density (**N**) (*n* = 8–10 per group). Scale bar: 10 μm. (**O**, **P**) Representative images (**O**) and quantification graphs (**P**) of c-Fos + cells in GCL (*n* = 6 per group), Scale bar: 500 μm. n. s., not significant

Stress and adult hippocampal neurogenesis interact reciprocally. The buffering effects of neurogenesis has been mostly focused on the roles of newly generated neurons in regulation of the existing mature granule neurons [4]. For example, stress activates mature GCs in ventral DG, and these stress-responsive neurons in this area are inhibited by adult-born neurons generated through hippocampal neurogenesis, leading to stress resilience [14]. In the present study, however, we showed that preservation of the NSC pool by prevention of CRS-induced autophagic death blocks activation of GC in the DG and rescues neuronal atrophy induced by CRS. Generation of new neuron from NSCs takes at least 4 weeks to mature [13]. Therefore, our findings uncovered that preservation of NSCs pool after CRS is required for normal hippocampal function.

In summary, our results suggest that maintenance of NSCs during CRS keeps hippocampal neuronal function intact through the neurogenesis-independent, direct regulation of hippocampal function.

## Methods

Methods can be found in Supplementary file 1.

## Electronic supplementary material

Below is the link to the electronic supplementary material.

Supplementary Material 1

## Data Availability

No datasets were generated or analysed during the current study.
