# Supplementary material for: Neural stem cell-specific deletion of Atg7 alleviates hippocampal dysfunction and neuronal alterations induced by chronic restraint stress
Source: Mol Brain. 2025 Mar 21;18:25. doi: 10.1186/s13041-025-01189-8 (PMC11927343; doi:10.1186/s13041-025-01189-8)
Supplement: Supplementary file 1 — Supplementary Material 1 [file 13041_2025_1189_MOESM1_ESM.docx]

**Methods**

**Animals and CRS procedure**

All procedures for the care and use of laboratory animals were approved by the Institutional Animal Care and Use Committee of DGIST. All animals were maintained in a specific pathogen free environment at the DGIST animal facility. *Atg7*^NSC^ cKO mice were produced by crossing *Nestin*-Cre/ERT2 transgenic mice with heterozygous *Atg7* floxed mice (Atg7fl/+), as previously described [1]. TAM was dissolved in 10% ethanol and 90% corn oil and administered by intraperitoneal injection at 150 mg/kg/day for 3 days at the age of 7 weeks to activate Cre. CRS started 7 days after the last administration of TAM. Each mouse of CRS group was placed in plastic restrainer from 11 AM to 2 PM daily for 14 days. Untreated group mice were deprived of food and water during restraint time.

**Reagents and antibodies**

Antibodies used for immunofluorescence staining were follows: SOX2 (mouse, 1:500, Abcam, ab79351), NeuN (mouse, 1:1000, Millipore, MAB377), KI67 (rabbit, 1:500, Abcam, ab1558), c-Fos (rabbit, 1:400, Santa Cruz, sc-52), Alexa Flouor 488 mouse IgG (mouse, 1:500, Jackson Laboratories, 715-545-151) and Alexa Fluor 555 rabbit IgG (rabbit, 1:500, INVITOGEN, A-31572).

**Behavior analyses**

Behavior analyses were conducted with 2 cohorts of mice with one for Y-Maze, OFT, FST and the other for SPT.

*Y-maze test*

The Y-maze consists of three arms that extends from a central platform at a 120° angle. Mice were placed in the center of the Y-maze and was explored the maze for 6 minutes. The arm alteration and total number of arms entered were recorded using Ethovision Observer (Noldus).

*Open field test (OFT)*

This test was conducted in a square-shaped arena (40 × 40 cm^2^) box. Mice were placed and allowed to explore the field for 20 minutes. The time spent in the center region (20 × 20 cm^2^) of the box and total distance moved were measured. Data were collected using Ethovision Observer (Noldus).

*Forced swimming test (FST)*

Mice were forced to swim in a 30 cm cylinder container filled with water at a temperature of 23-25℃ and a depth of 15 cm for 6 minutes. Immobility was analyzed for last 4 minutes by Ethovision Observer (Noldus).

*Sucrose preference test (SPT)*

Mice were habituated with distilled water and 1% sucrose water, respectively for 3 days. After adaptation, mice were deprived of water for 6 hours and then distilled water and 1% sucrose water were replaced for 12 hours to measure preference of sucrose. To avoid place preference, sucrose and water bottles were swapped during test session. The preference of sucrose was calculated as follows;

[sucrose water consumption x 100 / (distilled water consumption + sucrose consumption)]

**Immunohistochemistry (IHC) analysis**

Mice were deeply anesthetized by an injection of 2% avertin and perfused with phosphate-buffered saline (PBS), followed by 4% paraformaldehyde (PFA). Brains were taken out, post-fixed in 4% PFA for 12 hours and cryoprotected in 30% sucrose until they sank. Brains were frozen in OCT compound and cut into 40 μm thick coronal sections. Samples were kept in PBS and blocked for an hour (PBS + 1% bovine serum albumin + 0.5% Triton X-100) and then incubated with the primary antibodies at 4°C overnight and secondary antibodies for an hour at room temperature with shaker. We used LSM 780 or 800 confocal laser scanning microscope (Carl Zeiss) and fluorescence microscope (Nikon) to examine the samples. Stereological counting of the quantification of NSCs was conducted using Stereo Investigator software (MBF bioscience) with bregma -1.2 mm to -2.8 mm. The c-Fos+ cells were counted with bregma -2.02 mm to -2.98 mm in GCL.

**Golgi-Cox staining**

Golgi-Cox staining was conducted with Golgi-Cox OptimStainTM Kit (HITO Biotec Corp.) by according to the manufacturer’s instructions. Samples were cut into 150 μm thick coronal sections through vibratome. Neurons in CA3 regions (bregma -1.06 mm to – 2.80 mm) were analyzed using Nikon microscope.

**Statistical analysis**

Data were expressed as mean ± standard error of the mean (SEM) and significance was set at p < 0.05 (See each figure for details). Sample sizes were found within figure legends. For comparison groups, Two-way ANOVA multiple comparisons were used through Tukey statistical hypothesis. All statistical analyses were carried out using GraphPad Prism Software (version 8.0)

**Reference**

1. Komatsu, M., et al., *Loss of autophagy in the central nervous system causes neurodegeneration in mice.* Nature, 2006. **441**(7095): p. 880-884.
